# Diagnostic usefulness of histamine release test (HRT) and skin tests in IgE-mediated allergy to clavulanic acid

**DOI:** 10.1186/2045-7022-4-S3-O7

**Published:** 2014-07-18

**Authors:** Fernando Pineda, Adriana Ariza, Cristobalina Mayorga, Inmaculada Perez, Rosario Gonzalez-Mendiola, Natalia Blanca, Galicia Davila, Nieves Cabañes, Gabriela Canto, Jose Julio Laguna, Carlos Senent, Per Stahl Skov, Ricardo Palacios, Miguel Blanca

**Affiliations:** 1 DIATER Laboratories, Spain; 2 IBIMA, Regional University Hospital of Malaga, UMA, Allergy Unit, Spain; 3 Hospital de la Cruz Roja, Allergy Unit, Spain; 4 Hospital Infanta Leonor, Allergy Unit, Spain; 5 Hospital del Henares, Allergy Unit, Spain; 6 Hospital Virgen del Valle, Allergy Unit, Spain; 7 University Hospital of Odense, Dermatology, Denmark

## Background

Clavulanic acid (CLV) is an â-lactam antibiotic highly consumed in combination with amoxicillin (AX). In the last years, CLV-related hypersensitivity reactions have been reported, the diagnosis being mainly confirmed by skin and drug provocation tests (DPT)

## Aim

Evaluation of a group of patients with immediate hypersensitivity reactions to CLV using skin tests and *in vitro* tests such as direct and passive sensitization HRT

## Methods

Nineteen patients with an immediate hypersensitivity reaction after AX-CLV administration were evaluated. Skin tests were done with PPL, DM, AX and CLV (DIATER, Madrid, Spain). Controls (n=21) with proven AX-CLV tolerance were included in the context of a Spanish cross-sectional multicentric study including five hospitals. In vitro tests were performed by direct and passive sensitization HRT (Reflab, Copenhagen, Denmark) using AX and CLV at different concentrations. Tolerance to AX was assessed by DPT.

## Results

Patients' clinical manifestations consisted of anaphylaxis (n=7), urticaria (n=9) or angioedema/urticaria (n=3). All patients had skin test negative to PPL, DM, and AX and positive to CLV (2 by prick test at 20 mg/mL, and 17 by intradermal testing at 0.5 mg/mL (n=2), 5 mg/mL (n=9) and 20 mg/mL (n=6). AX DPT test was negative, thus confirming a CLV selective reaction. Skin testing was negative in all controls. Direct HRT sensitivity and specificity were 77.8% and 66.7%, respectively, and for passive sensitization HTR, sensitivity and specificity were 78.9% and 76.2%, respectively. AX HRT was negative in all patients

## Conclusions

High sensitivity and specificity rates shown by passive sensitization HRT support that CLV-related hypersensitivity reactions are IgE-mediated. Also of interest, skin tests are becoming a useful method for the diagnosis of this condition.

